# Supplementary material for: Phosphorylation of CaMK and CREB-Mediated Cardiac Aldosterone Synthesis Induced by Arginine Vasopressin in Rats with Myocardial Infarction
Source: Int J Mol Sci. 2022 Dec 1;23(23):15061. doi: 10.3390/ijms232315061 (PMC9738971; doi:10.3390/ijms232315061)
Supplement: Supplementary file 1 [file ijms-23-15061-s001.zip › ijms-2038863-supplementary.pdf]

International Journal of *Molecular Sciences*

Supplementary

Phosphorylation of CaMK and CREB-Mediated Cardiac Aldosterone Synthesis Induced by Arginine Vasopressin in Rats with Myocardial Infarction

Yuan-Sheng Zhai [1,2,†], Jie Li [1,2,†], Longyun Peng [1,2], Guihua Lu [1,2], and Xiuren Gao [1,2,*]

1 Department of Cardiology, the First Affiliated Hospital, Sun Yat-Sen University, Guangzhou 510800, China
2 Key Laboratory on Assisted Circulation, Ministry of Health, Guangzhou 510800, China
† These authors contribute equally to the work.
* Correspondence: gaoxr@mail.sysu.edu.cn; Tel.: +86-020-82394178

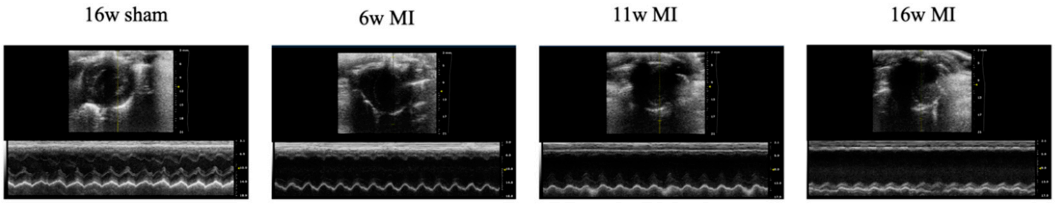

Figure S1. Representative M-mode echocardiographic images of rats. 16w sham, 16-week post-sham-operation group; 6w MI, 6-week post-infarction group; 11w MI, 11-week post-infarction group; 16w MI, 16-week post-infarction group .

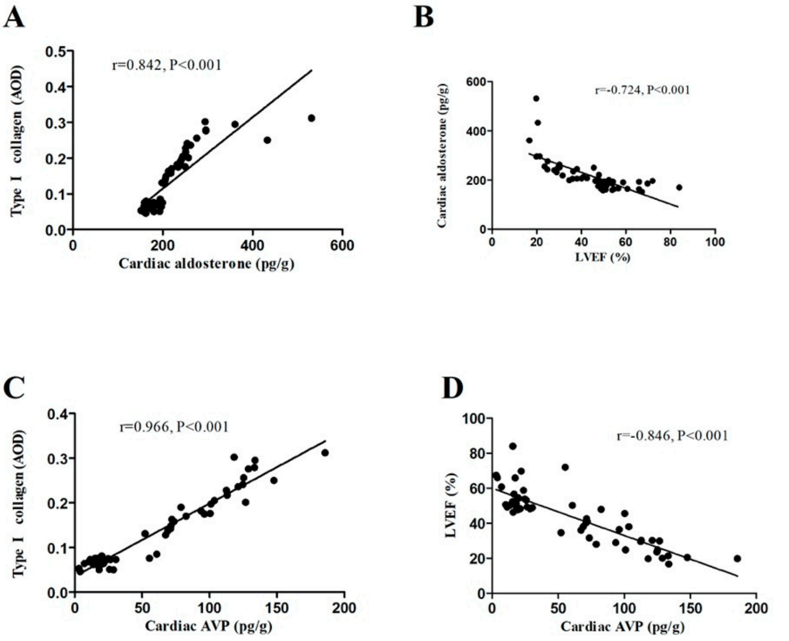

Figure S2. Correlation analyses among the rats. LVEF, left ventricular ejection fraction. A: Correlation analysis between aldosterone and type I collagen expression. B: Correlation analysis between aldosterone and LVEF. C: Correlation analysis between AVP and type I collagen expression. D: Correlation analysis between AVP and LVEF.
